# Beyond taxonomic diversity patterns – investigating how α and β components of macrophyte functional diversity respond to environmental gradients in lotic ecosystems of Greece

**DOI:** 10.3389/fpls.2023.1204383

**Published:** 2023-09-08

**Authors:** Konstantinos Stefanidis, Anthi Oikonomou, Georgios Dimitrellos, Dionysios Tsoukalas, Eva Papastergiadou

**Affiliations:** ^1^ Department of Biology, University of Patras, Patras, Greece; ^2^ Hellenic Centre for Marine Research, Institute of Marine Biological Resources and Inland Waters, Attiki, Greece

**Keywords:** aquatic macrophytes, functional richness, functional beta diversity, generalized dissimilarity models, rivers

## Abstract

In addition to quantifying the taxonomic diversity of aquatic communities, understanding the patterns of alpha functional diversity (α-diversity) and exploring changes in functional dissimilarity (β-diversity) can improve our understanding on how ecosystems respond to environmental changes. In this study, we quantified functional alpha (α) and beta (β) diversity of macrophytic assemblages from river sites in Greece and then, examined relationships with water quality parameters and hydromorphological factors. We assigned 6 traits (Ellenberg nutrients indicator, Ellenberg light indicator, growth form, leaf size, leaf type, fruit size) to a total of 36 hydrophyte species and calculated three indices of functional diversity (functional richness, functional dispersion and functional evenness). We also estimated the total β-functional diversity and its’ main components, turnover and nestedness. To assess the effects of water quality (including physical and chemical variables) we used Generalized Additive Models (GAM) for alpha functional diversity indices and Generalized Dissimilarity Models (GDM) for beta functional diversity. We performed Kruskal-Wallis tests and permutational multivariate analysis of variance (PERMANOVA) to search for significant variations of α- and β-diversity among the hydromorphological factors. Our results showed that macrophyte growth form and light preference were important trait characteristics that explained a large share of the total variance of functional composition. We also found relatively low taxonomic and functional richness, whereas taxonomic and functional dissimilarity were mostly attributed to species turnover, which expresses the changes in taxonomic and functional composition. We found significant relationships between functional dispersion and functional evenness with pH and oxygen saturation, whereas functional dissimilarity was driven only by geographic distance, although the GDM explained a small portion of the total variance. Functional richness, dispersion and evenness were significantly higher at systems with fine substrates and deep waters with low or high flow compared to systems with coarser substrates and riffle habitats. We also found significant variation in functional dissimilarity among the hydromorphological factors, although much of the total variance remained unexplained. Overall, our study highlights the importance of considering the functional diversity of aquatic plant assemblages within the frame of freshwater monitoring and conservation plans.

## Introduction

Aquatic macrophytes provide numerous functions in lotic ecosystems. For instance, plants that grow within the channel and along the banks are known to mediate nutrient and sediment transport from the land into the watercourse (Valkama et al., 2019; Walton et al., 2020), while at the same time they stabilize the channel and the banks preventing erosion. Most importantly, aquatic plants can provide foraging and reproduction habitats for fish, amphibians and invertebrates (Lind et al., 2019; Cole et al., 2020). They can also influence the physical, chemical and flow characteristics within the channel (Gurnell, 2015; Preiner et al., 2020), which in turn may affect fish and invertebrate communities.

Because of their importance for stream and riverine ecosystems, aquatic macrophytes have been widely used as indicators of ecosystem health and ecological integrity (Aguiar et al., 2014; Rodrigues et al., 2019; Szoszkiewicz et al., 2020). Aquatic macrophytes are one of the four biological quality elements that are used for the assessment of ecological status of streams and rivers of Europe, following the implementation of the Water Framework Directive 2000/60, and several national assessment approaches have been developed by the EU Member States (Birk and Willby, 2010). Most ecological assessment schemes consider the composition of aquatic macrophytic communities (Szoszkiewicz et al., 2020; Stefanidis et al., 2022) since the increased occurrence and abundance of certain plants is related with environmental factors that indicate anthropogenic degradation of aquatic ecosystems (e.g. eutrophication and hydromorphological alteration) (Szoszkiewicz et al., 2014; Stefanidis and Papastergiadou, 2019; Manolaki et al., 2020; Stefanidis et al., 2021). However, there are studies that have shown that the responses of macrophytes to environmental gradients can be complex and difficult to decipher (Steffen et al., 2014; O’Hare et al., 2018; Son et al., 2018; Gyosheva et al., 2020). Thus, freshwater ecologists have shown increased interest in studying multiple facets of aquatic biodiversity, including aquatic macrophytes (Fu et al., 2014a; Schneider et al., 2015; Alahuhta et al., 2017; Elo et al., 2018; Son et al., 2018; Stefanidis et al., 2019).

The functional diversity which Tilman (2001) defined as ‘those components of biodiversity that influence how an ecosystem operates or functions’, has emerged as a facet of biodiversity and a step beyond species richness. It has become a powerful tool to link community composition to ecosystem properties and then to ecosystem services by quantifying the value and range of functional characteristics and thus ecosystem functioning (Díaz et al., 2007). In addition, the impact of the global loss of biodiversity is increasingly attributed to the loss of functional rather than taxonomic groups (Bellwood et al., 2002). Further, species are not equal in their effects on ecosystem functioning since their functional traits matter to ecosystem processes (Mouchet et al., 2010). The study of trait distributions can be used as a more powerful conceptual model for understanding broad-scale patterns in assemblage structure since organisms with similar traits will share similar niche requirements and will select the same habitat (Olden et al., 2010).

Besides species richness (alpha diversity) and changes in species composition among communities (beta diversity), investigating patterns of functional alpha (α) and beta (β) diversity may provide invaluable information and a better understanding on how environmental gradients affect aquatic biodiversity processes (Zhang et al., 2018; Wang et al., 2021). Considering that assembly processes influencing natural communities may differ depending on the spatial scale considered, separating functional diversity in within-community (α) and among-community (β) components will improve the detection of all processes influencing community assembly. Furthermore, trait-based approaches are more likely to indicate an early response to environmental change than community-based approaches because a change in the functional structure is easier to detect than a change to community composition (Degen et al., 2018). In addition, such approaches can be applied to all species regardless of geographic region and location (Dolédec et al., 2006).

Previous studies have linked morphological and life history traits of aquatic plants with ecological and biogeochemical processes showing the response of aquatic plant functional structure to eutrophication and other environmental changes (Fu et al., 2014b; Baattrup-Pedersen et al., 2016; Stefanidis and Papastergiadou, 2019). Therefore, exploring the patterns of functional trait composition in relation to environmental change is crucial for a better understanding of the response of aquatic communities to pressure and identifying key trait characteristics that can serve as indicators of anthropogenic pressures. Although there is a general consensus that taxonomic diversity is shaped by environmental filters, including anthropogenic changes (Bornette and Puijalon, 2011; Dybkjaer et al., 2012), there are relatively few studies that have explored the patterns of functional α- and β- diversity of aquatic macrophytes in lotic ecosystems.

The main objective of this study is to investigate the patterns of functional alpha and beta diversity of aquatic macrophytic assemblages in Greek lotic ecosystems and to assess whether these patterns are subject to environmental change. Our hypotheses are that a) indices of a- functional diversity decline with increased levels of nutrient pollution and degraded water quality, b) the change of functional compositional structure increases with water quality impairment and c) α- and β- functional diversity show significant variations among different types of hydromorphological conditions and different degrees of hydromorphological modification.

## Material and methods

### Macrophyte samplings

**Figure 1:**
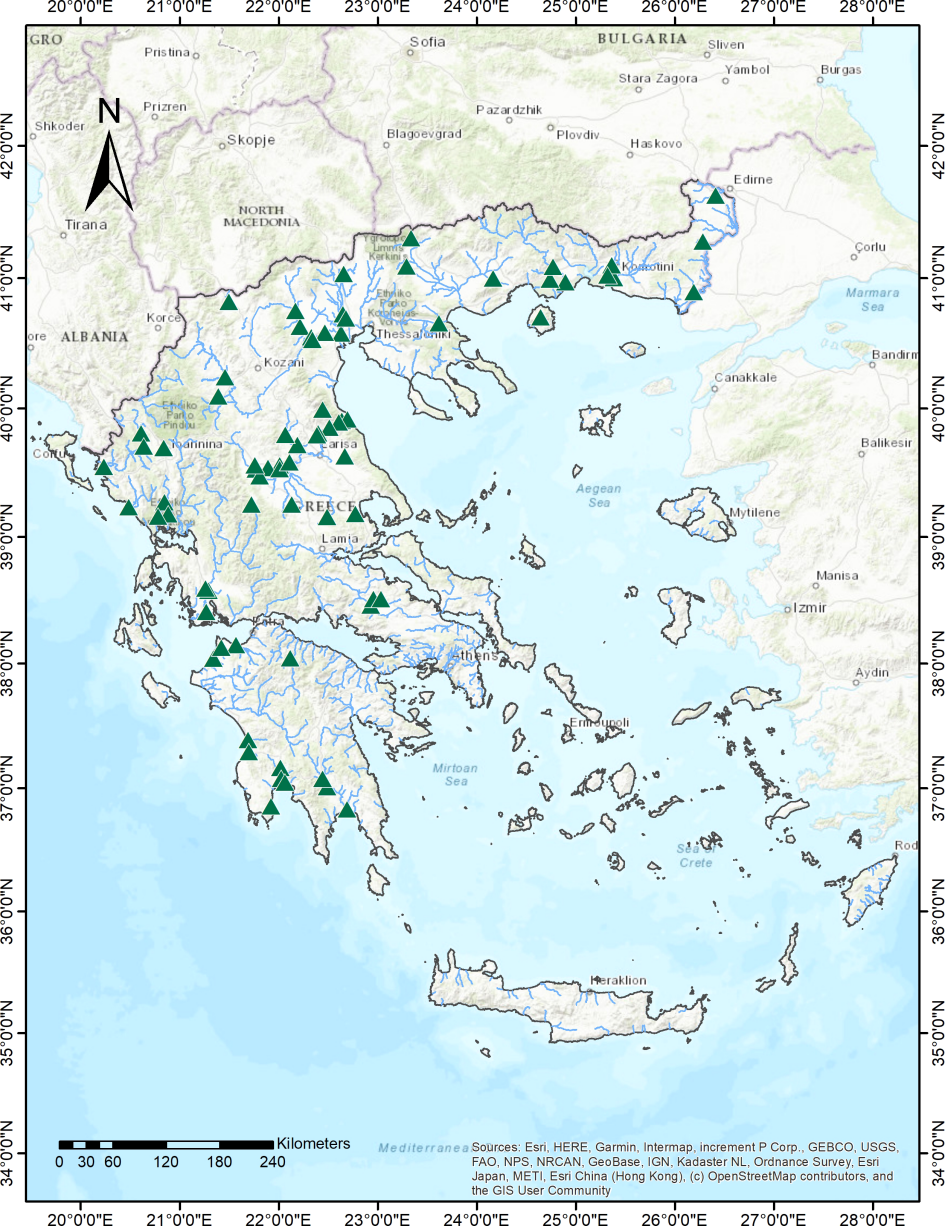
Map showing the location of sampling sites (N=74) of the National Monitoring network, across running waters of mainland Greece.

Field samplings were conducted in summer of 2021 and 2022 as part of the ecological monitoring program for the assessment of the ecological status of rivers of Greece in line with the EU Water Framework Directive (WFD) (Skoulikidis et al., 2021). We used presence-absence data of hydrophytes, plants that grow exclusively in water, from 74 river sites that belong to the national monitoring network for the ecological quality assessment of inland waters. The sampled sites extend across several biogeographic regions of Greece ranging from 37^°^N to 41°N and 20°E to 25°E and at altitudes spanning from sea level to 765 m a.s.l (**Figure 1**).

Macrophytes were sampled in accordance with national protocols harmonized with European standards (CEN, 2003; CEN, 2006). Sampling was conducted by wading into the water, following a zigzag pattern upstream for a 100 meter-long section of the river channel. Unidentified specimens were collected and transferred at the laboratory for identification.

### Environmental parameters

We used geographical variables (latitude, longitude and altitude), physical and chemical parameters (electrical conductivity, total dissolved solids, pH, dissolved oxygen concentration, dissolved oxygen saturation and biochemical oxygen demand, nitrate, nitrite, ammonium, total nitrogen and total phosphorus) and hydromorphological characteristics in order to identify links between facets of plant diversity and environmental characteristics. Water physicochemical parameters and water samples for the determination of nutrients were obtained during samplings that were conducted during the same period with the macrophyte surveys. Hydromorphological features (e.g. type of channel substrate, effects of hydrological and morphological modifications, bed stability, channel shade) were assessed during the plant sampling according to Stefanidis et al. (2022). A description of the environmental variables that were considered in this study is listed in **Table 1**. For further details on field samplings, characteristics of river reaches, macrophyte identification and quantification of physicochemical and geomorphological variables see our publications (Stefanidis et al., 2021; Papastergiadou, 2022; Stefanidis et al., 2022).

**Table 1:** Details and brief description of the environmental variables that were considered in this study.

Category	Variable name	Description	Variable type	Mean value
Water quality/physicochemical	EC	Electrical Conductivity [μS/cm]	Numerical continuous	693.52
	pH	Sorensen scale	Numerical continuous	7.91
	DO	Concentration of dissolved oxygen [mg/l]	Numerical continuous	7.59
	BOD	Biochemical oxygen demand [mg/l]	Numerical continuous	7.13
	Nitrate	Nitrate concentration in the water [mg/l NO_3_^-^]	Numerical continuous	1.27
	Nitrite	Nitrite concentration in the water [mg/l NO_2_^-^]	Numerical continuous	0.04
	Ammonium	Ammonia concentration in the water [mg/l NH_4_^+^]	Numerical continuous	0.39
	TN	Total Nitrogen [mg/l N]	Numerical continuous	1.92
	Phosphate	Concentration of Orthophosphates in the water [mg/l PO_4_^3-^]	Numerical continuous	0.16
	TP	Concentration of total phosphorus in the water [mg/l P]	Numerical continuous	0.17
	TDS	Concentration of total dissolved solids [mg/l]	Numerical continuous	366.21
Hydromorphological/habitat	Channel substrate	Prevailing channel substrate, three levels: Fine (<2mm), medium (2 – 64 mm), coarse (>64 mm)	Ordinal factor	NA
	Bed stability	Stability of riverbed, four levels: Solid (e.g. bedrock), stable, unstable, soft (e.g. mud)	Ordinal factor	
	Shade	Channel shade, three levels: Absence of shade, semi-continuous shade, full shade	Ordinal factor	
	Habitats	Type of river habitat: Pool, riffle, run, slack	Nominal factor	
	Land Uses	Type of prevailing land use within the adjacent area, four levels: Artificial, Agriculture, Natural, Wetland	Nominal factor	
	Channel profile alteration	Degree of channel profile modification present at the site/cross section alteration	Ordinal factor	
	Morphology alteration	Degree of the morphological modification of the channel present at the site	Ordinal factor	
	Habitat alteration	Alteration of instream habitats	Ordinal factor	
	Stream hydrology alteration	Degree of the hydrological alteration present at the site	Ordinal factor	
	Water abstraction	Influence of water abstraction at the site	Ordinal factor	
	Dykes (flood protection)	Influence of dykes at the site	Ordinal factor	

NA, not applicable.

### Trait-based analysis and calculation of functional diversity indices

Patterns of functional composition and diversity of aquatic macrophytes were investigated following a methodological framework that is based on the construction of multidimensional functional space using community and trait data (Magneville et al., 2022). First, we created a matrix with presence-absence data of macrophyte species for the 74 river sites. Then, we constructed a matrix with functional community characteristics based on six nominal and ordinal plant traits allocated to a total of 36 hydrophyte species (**Table 2**). The traits that we used were the Ellenberg indicator values for nitrogen and light preference (Tichý et al., 2023), the life-form according to Wilby et al. (Willby et al., 2000), the leaf size and fruit size classified into three categories (small, moderate, and large) (Willby et al., 2000), and the leaf type classified into three types (entire, capillary and tubular) (Willby et al., 2000). **Table 3** includes a list with the allocated traits that we used.

**Table 2:** List of aquatic macrophyte species considered in the present study.

Code	Name	Code	Name
Ali.lan	*Alisma lanceolatum* With.	Oen.aqu	*Oenanthe aquatica* L.
Ali.pla	*Alisma plantago-aquatica* L.	Per.amp	*Persicaria amphibia* (L.) Gray
Api.nod	*Apium nodiflorum* (L.) Lag.	Pot.cri	*Potamogeton crispus* L.
Azo.fil	*Azolla filiculoides* Lam.	Pot.nat	*Potamogeton natans* L.
Ber.ere	*Berula erecta* (Huds.) Coville	Pot.nod	*Potamogeton nodosus* Poir.
But.umb	*Butomus umbellatus* L.	Pot.per	*Potamogeton perfoliatus* L.
Cal.sta	*Callitriche stagnalis* Scop.	Ran.cir	*Ranunculus circinatus* Sibth.
Cer.dem	*Ceratophyllum demersum* L.	Ran.flu	*Ranunculus fluitans* Lam.
Cer.sub	*Ceratophyllum submersum* L.	Ran.tri	*Ranunculus trichophyllus* Chaix ex Vill.
Gly.flu	*Glyceria fluitans* (L.) R.Br.	Ror.amp	*Rorippa amphibia* (L.) Besser
Hyd.mor	*Hydrocharis morsus-ranae* L.	Sal.nat	*Salvinia natans* (L.) All
Jun.Bul	*Juncus bulbosus* L.	Spa.eme	*Sparganium emersum* Rehmann
Lem.gib	*Lemna gibba* L.	Spa.ere	*Sparganium erectum* L.
Lem.min	*Lemna minor* L.	Stu.pec	*Stuckenia pectinata* (L.) Böerner
Men.aqu	*Mentha aquatica* L.	Tra.nat	*Trapa natans* L.
Myr.alt	*Myriophyllum alterniflorum* DC.	Val.spi	*Vallisneria spirali*s L.
Myr.spi	*Myriophyllum spicatum* L.	Ver.ana	*Veronica anagalis-aquatica* L.
Nas.off	*Nasturtium officinale* W.T.Aiton	Ver.bec	*Veronica beccabunga* L.
Nup.lut	*Nuphar lutea* (L.) Sm.	Zan.pal	*Zannichellia palustris* L.
Nym.alb	*Nymphaea alba* L.		

**Table 3:** Overview of the aquatic macrophyte traits used in the present study.

Trait Code	Trait Name	Category	Values
EIV N	Ellenberg N—nutrients preference	Ecological preference	1: low nutrients, 5= intermediate levels of nutrients, 9= rich conditions of nutrients
EIV L	Ellenberg L—light preference	Ecological preference	1 = deep shade, 5 = semi shade, 9 = full light
GF	Growth form	Life form	AEL: anchored with emergent leaves, AFL: anchored with floating leaves, ASUB: anchored submerged plants, FFSUR: free floating on surface, FFSUB: free floating submerged
LS	Leaf size	Morphology	SMALL: ≤ 1cm^2^, MODERATE: 1–20 cm^2^, LARGE: ≥ 20 cm^2^
FS	Fruit size	Morphology	SMALL: ≤ 1cm^2^, MODERATE: 1–20 cm^2^, LARGE: ≥ 20 cm^2^
LT	Leaf type	Morphology	ENT: entire, CAP: capillary, TUB: tubular

A functional distance matrix that contains the functional distances for each pair of species was calculated using the Gower distance, since all traits are categorical. Then, a hierarchical cluster analysis was conducted on the distance matrix to obtain groups of plants with similar functional assemblages. Following Kelley et al. (1996), we employed the Kelley–Gardner–Sutcliffe penalty function (KGS) to identify distinct clusters of the dendrogram. This method maximises differences between groups and cohesiveness within groups. The minimum of the KGS function corresponds to the optimal number of clusters. Functional space was then constructed based on the functional dissimilarity matrix using a principal coordinates analysis (PCoA) and functional diversity indices were calculated using the species coordinates on the first three principal components (Maire et al., 2015). Correlations between traits and the functional axes were identified with a Kruskal-Wallis test to help understand how plant groups are distributed across the functional space with regard to their trait composition.

Concerning the functional indices, Mouillot et al. (Mouillot et al., 2013) has proposed the use of several indices that act complementary and can provide useful insights about the functional community structure. Here we calculated three of these indices. Functional dispersion, which shows the deviation of species traits values from the center of the functional space filled by the assemblage, the functional richness which represents the amount of functional space occupied by a species assemblage, and the functional evenness which corresponds to how regularly species abundances are distributed in the functional space (Mason et al., 2005; Mouillot et al., 2013).

Beta (β) diversity was assessed as differences between all pairs of sites using the Sørensen index (βsor). The taxonomic and functional β dissimilarities are consistent with two additive components: the turnover component (replacement of species or functional space not shared by communities) and the nestedness-resultant component (difference in species or functional space filled by communities) (Baselga, 2010; Villéger et al., 2013). The turnover (βsim) and nestedness (βsne) components were quantified in accordance with the β-diversity partitioning framework proposed by Baselga (2010) and Villéger et al. (2013). The indices of β-diversity were calculated using the R package betapart (Baselga and Orme, 2012). These indices range from 0 to 1, where higher values indicate greater dissimilarities among sites.

By examining both taxonomic and functional β-diversity we intended to draw useful conclusions about the changes in taxonomic and functional composition and to associated them further with environmental characteristics. For the calculation of all functional diversity indices, we used the package “mFD” in R environment (Magneville et al., 2022). The correlations between taxonomic and functional β-diversity as well as between their respective components were tested using Mantel permutational tests.

### Functional diversity and environmental parameters

We used Generalized Additive Models (GAM) to investigate the relationships between the alpha functional diversity indices, taxonomic diversity (species richness) and the water quality predictors (physical and chemical). GAMs have been commonly used in ecology for fitting non-linear relationships between species and environmental predictors (Guisan et al., 2002; Leathwick et al., 2006). Models were fitted with the “mgcv” package in R environment (Wood, 2020) using cubic smoothing splines. In order to assess the variation of alpha diversity indices among the levels of hydromorphological factors, we conducted Kruskall-Wallis tests.

Prior to the model fitting, predictors were tested for collinearity by calculating the variance inflation factor (VIF) with the vifstep function of the “usdm” package (Naimi et al., 2014) in R environment. Environmental variables with VIF > 3 were excluded from further analysis (Vittinghoff et al., 2012). The other variables were used for fitting full models with functional richness, functional dispersion and functional evenness as response variables. Then, using the dredge function from package “MuMIN” (Barton, 2020), the model with the lowest Akaike information criterion (AICc) value was selected as the final model.

Furthermore, we calculated Moran’s coefficients based on the geographical coordinates of the sites, to evaluate the spatial autocorrelation in each final model. Calculations were made with R package “ape” (Paradis and Schliep, 2019).

To investigate how β-diversity changes across water quality gradients, we used Generalized Dissimilarity models. Generalized Dissimilarity Models (GDMs) model the dissimilarity in species composition as a function of environmental and geographical parameters using dissimilarity matrices (Fitzpatrick et al., 2013). In our analysis, we used the default three I-spline basis functions per predictor was used and we plotted the I-splines to visually assess how magnitudes and rates of the total functional dissimilarity, functional turnover and nestedness change along the environmental gradients. The variable importance and significance of each environmental predictor were estimated based on matrix permutation. Specifically, the environmental data were permutated 50 times and for each permutated matrix a new model was fitted. The model significance was estimated by comparing the global deviance of the GDM fit to un-permutated data with that of permutated data. Then the same process was repeated for each predictor separately to assess the variable significance and importance. The GDMs were fitted with the package “gdm” in R environment (Fitzpatrick et al., 2020). Finally, we conducted a permutational multivariate analysis of variance (PERMANOVA) to assess whether functional dissimilarity differs among levels of hydromorphological factors. PERMANOVA was run with the adonis2 function of the “vegan” package in R environment.

## Results

### Patterns of trait composition

**Figure 2:**
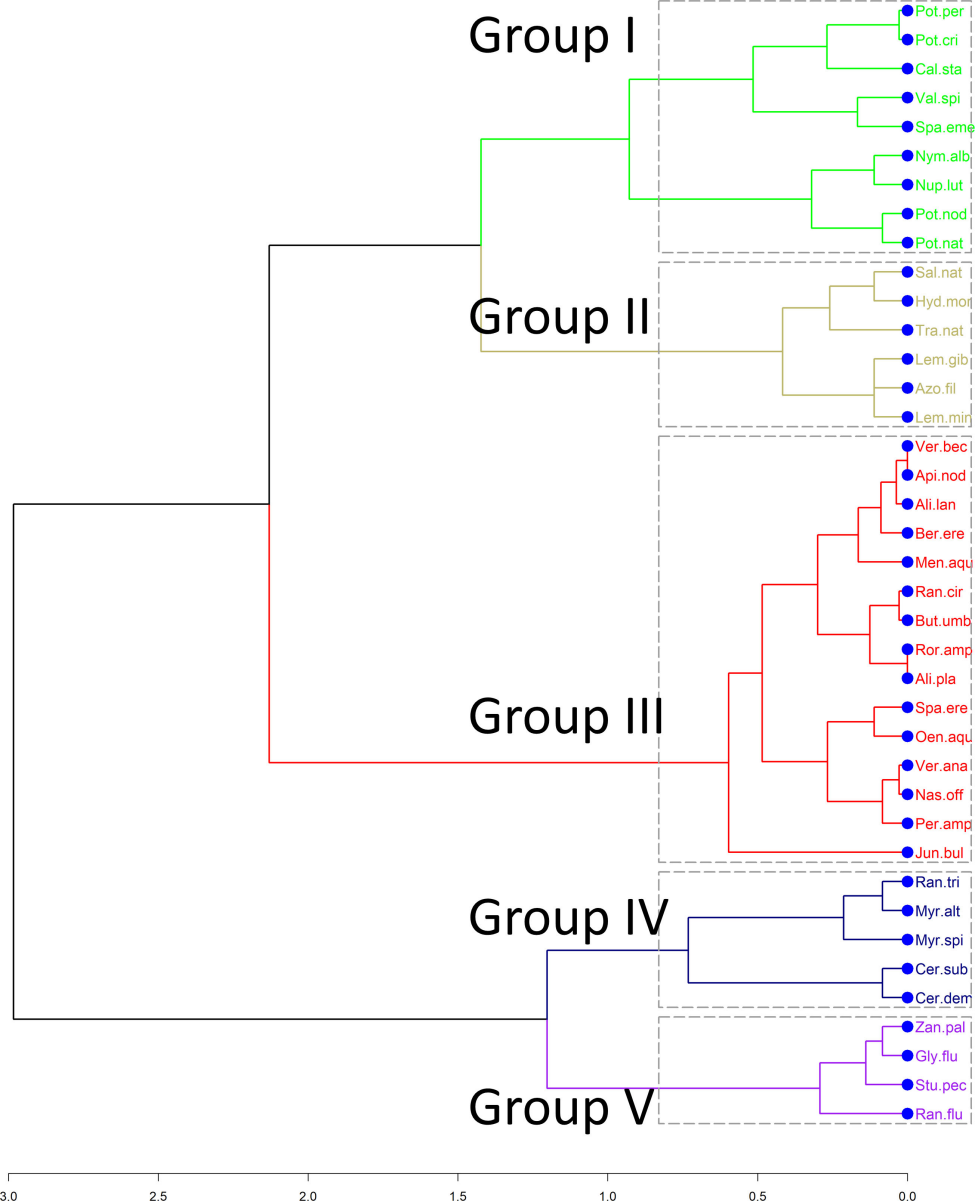
Dendrogram obtained from hierarchical clustering based on the species distances. Colors correspond to the five functional groups (I, II, III, IV, V) derived after the calculation of the Kelley-Gardner-Sutcliffe penalty.

The hierarchical cluster analysis based on the functional distance matrix revealed five distinct groups of plants (**Figure 2**). The first group (I) consists of nine species of macrophytes, four of which are rooted floating leaved, another four are rooted submerged and one (*Sparganium emersum*) can be found with either emergent leaves or floating on the surface. The second (II) functional group of macrophytes consists of four species that are exclusively free-floating and *Trapa natans* which is a floating-leaved plant usually anchored at the sediment. The third group includes emergent macrophytes growing their stems and leaves above the water surface (e.g. *Alisma plantago-aquatica, Mentha aquatica, Veronica anagalis-aquatica*). The two remaining groups (IV, V) include submerged species that seem to vary because of their leaf type. Group IV consists of plants that are fine-leaved (e.g. *Myriophyllum spicatum, Ranunculus trichophyllus, Ceratophyllum demersum*), whereas Group V is characterized by a mix of aquatic macrophytes that can have submerged leaves (fine-leaves or entire leaves), floating leaves or both.

**Figure 3:**
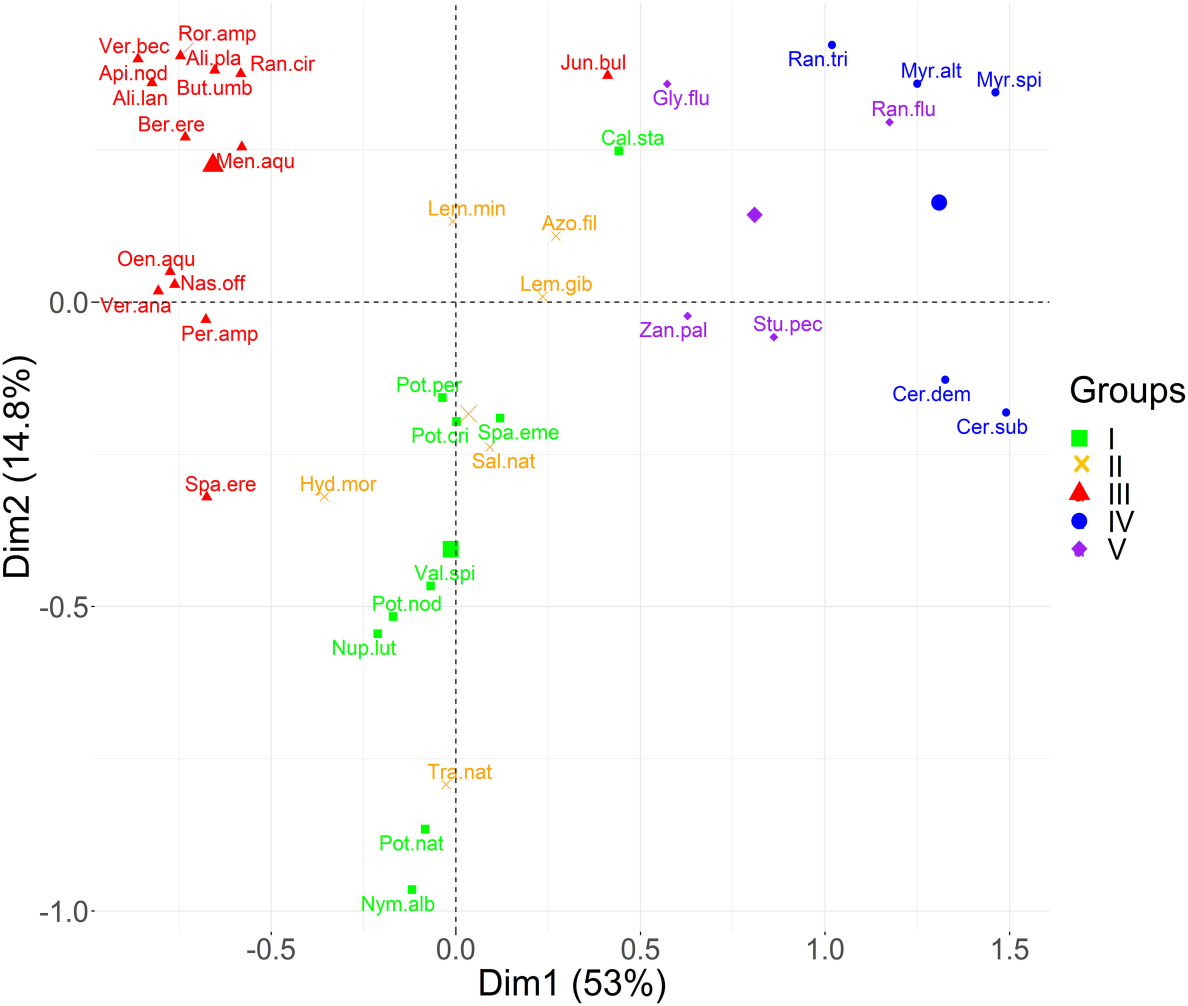
Principal component analysis plot of the species scores for the first two components. Colors correspond to the five functional groups (I, II, III, IV, V) derived after the calculation of the Kelley-Gardner-Sutcliffe penalty.

**Figure 4:**
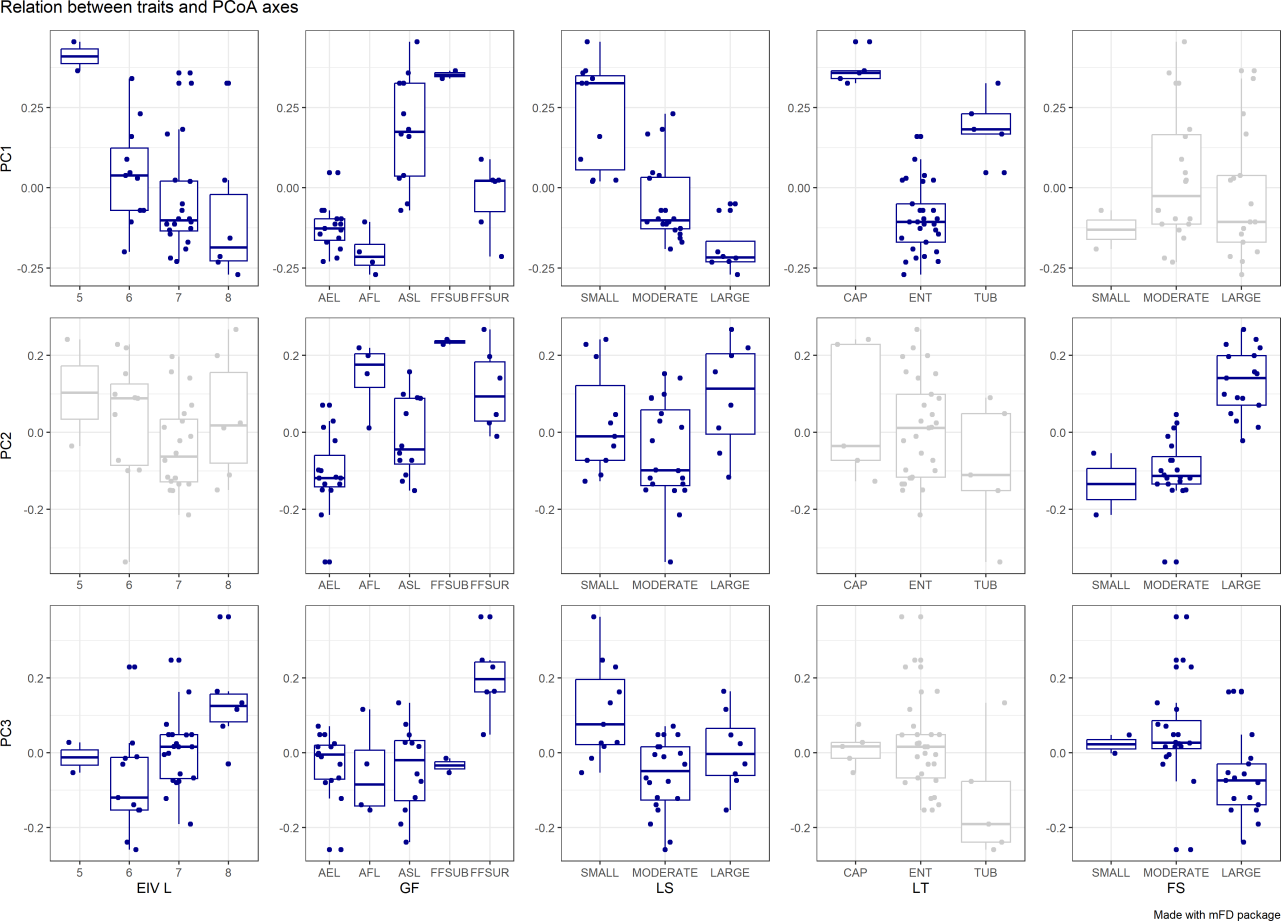
Boxplots show the relationship of the macrophyte species traits (EIV L: Ellenberg Light, GF: Growth Form, LS: Leaf Size, LT: Leaf Type, FS: Fruit Size) with the first three principal components. Dark blue plots indicate significant relationships (p ≤ 0.05).

The first two principal components of the PCA performed on trait data accounted for more than 60% of the variance among species characteristics (**Figure 3**). The first component (PC1) explained 53% of the total variance of the data, while the PC2 contributed with another 15%. Based on the boxplots of **Figure 4**, the first PC is mostly related with the traits Ellenberg light (EIV L), growth form (GF), leaf type (LT) and leaf size (LS), whereas the second PC is correlated significantly with Ellenberg light (EIV L), growth form (GF) and fruit size (FS), (**Figure 4**). Hence, the position of aquatic plants and functional groups along the two axes can indicate a strong affinity with specific functional traits and reflect specific ecological preferences. Plants that are more shade tolerant (lower values of EL) are positioned across the right part of the PCA plot (Groups IV and V) whereas plants that prefer good light conditions are clustered across the left part of the PC1 (mostly Group III), **Figure 3**. Macrophytes from Groups IV and V are also characterized by small and fine leaves opposed to Group III which consists of plants that have larger leaves and can be either emergent or floating-leaved. The position of the macrophytes across PC2 appears to relate mostly with the fruit size and the floating-leaved growth form, since macrophytes with floating leaves and larger fruits are positioned at the top part of the plot (**Figures 3**, **4**).

### Alpha (α-) and beta (β-) functional diversity patterns

**Figure 5:**
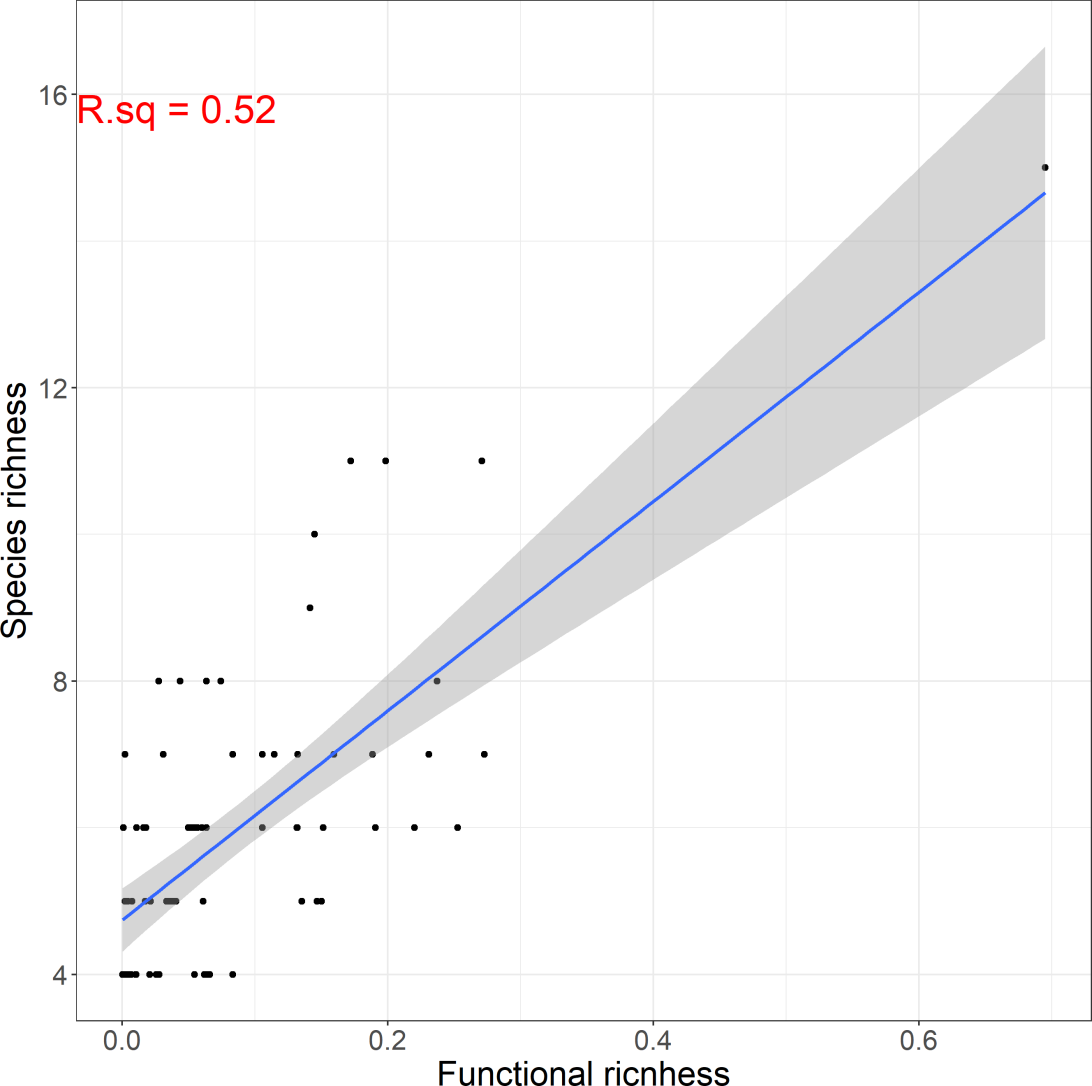
Linear relationship between functional and taxonomic macrophyte species richness among sites.

Species richness was relatively low, ranging by a minimum of 4 to a maximum of 15 species per site. Alpha functional diversity was described by three indices: functional richness, dispersion and evenness. The average value of functional richness for all sites was 0.11, with a maximum of 0.39. Functional dispersion and evenness were higher ranging from 0.23 to 0.6 and 0.38 to 0.71 respectively. Additionally, we found that functional diversity was positively correlated (r=0.72) to taxonomic diversity (**Figure 5**). However, we have to note here that this result was influenced by a community found in a relatively pristine site, which showed remarkably higher species and functional richness than most communities. Excluding this community from our dataset yields a correlation coefficient r=0.59 (significant at p-value ≤ 0.001), which is still relatively high, but considerably lower than 0.72.

Taxonomic β diversity ranged from 0.08 to 1 with a mean value of 0.72 (± 0.19). The turnover (βsim, 0.65± 0.23) was higher than the nestedness-resultant component (βsne, 0.07). Functional dissimilarity based on Sørensen among the aquatic plant communities was high (0.85 ± 0.20), with the functional turnover (i.e. the communities host different functional strategies) accounting for 62% (0.62 ± 0.34) and dissimilarity due to difference in functional richness (nestedness) accounting for 23% of the total variation.

Functional β diversity was significantly correlated with taxonomic β diversity (Mantel test, r = 0.48, p < 0.001), with functional turnover and taxonomic turnover being also strongly correlated (r = 0.54, p < 0.001). Nestedness-resultant components of taxonomic and functional β diversity were also correlated (r = 0.48, P < 0.001).

### Relationships between functional diversity indices and environmental descriptors

**Figure 6:**
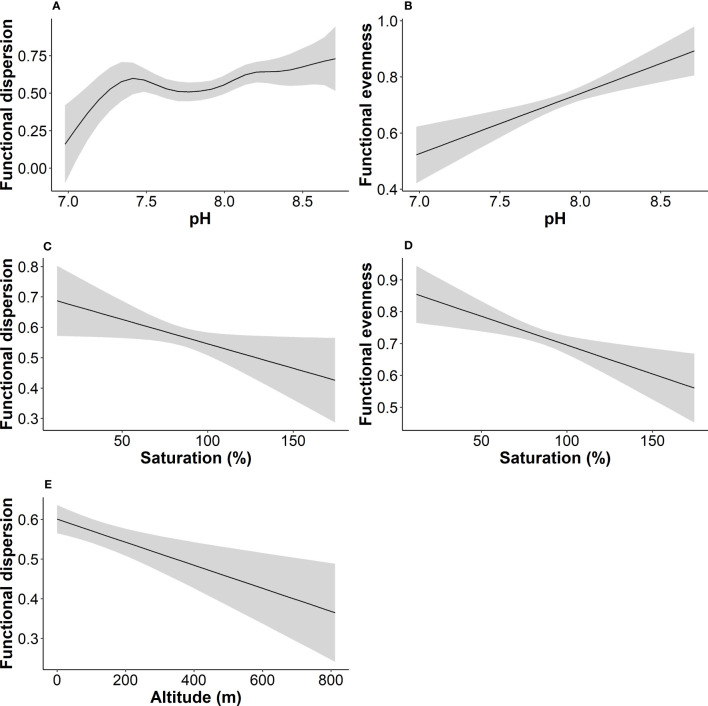
Fitted functions of functional dispersion **(A, C, E)**, and functional evenness **(B, D)** to partial effects of statistically significant environmental variables. Line represents GAM smoothing curve while the grey area depicts the standard error.

We tested for relationships between α functional diversity indices and water quality variables with the use of GAMs (**Figure 6**). The results of the best model fits showed that a relatively small share of variance of functional dispersion and functional evenness (25.6 and 25.3%) was explained by environmental variables. For functional richness the percentage of variance explained by the environment was even lower (approximately 11%). The best model for functional dispersion retained four predictors, with altitude, pH and oxygen saturation being significant (**Table 4**). For functional evenness the model retained five predictors with pH and oxygen saturation being significant (**Table 4**). The best model for functional richness, which had the lowest R^2^ among the three functional indicators, retained two variables (altitude and BOD) with neither being statistically significant.

**Table 4:** Summary of GAMs fitted to the functional richness, functional dispersion, functional evenness and species richness.

Response variable	Adj.R^2^	% Deviance explained	Retained explanatory variables	Significance (p value)
Functional richness	0.11	14.9	BOD	0.075
			Altitude	0.062
Functional dispersion	0.26	34.1	Altitude	0.001
			pH	0.020
			Ammonium	0.060
			Oxygen saturation	0.042
Functional evenness	0.25	31.8	Turbidity	0.076
			Oxygen saturation	0.004
			pH	<0.001
			BOD	0.198
			TDS	0.275
Species richness	0.08	11.5	Ammonium	0.054
			TDS	0.069

The retained explanatory variables for each model along with p-values are shown. Adjusted R^2^ and percentage of deviance explained are also shown.

**Figure 7:**
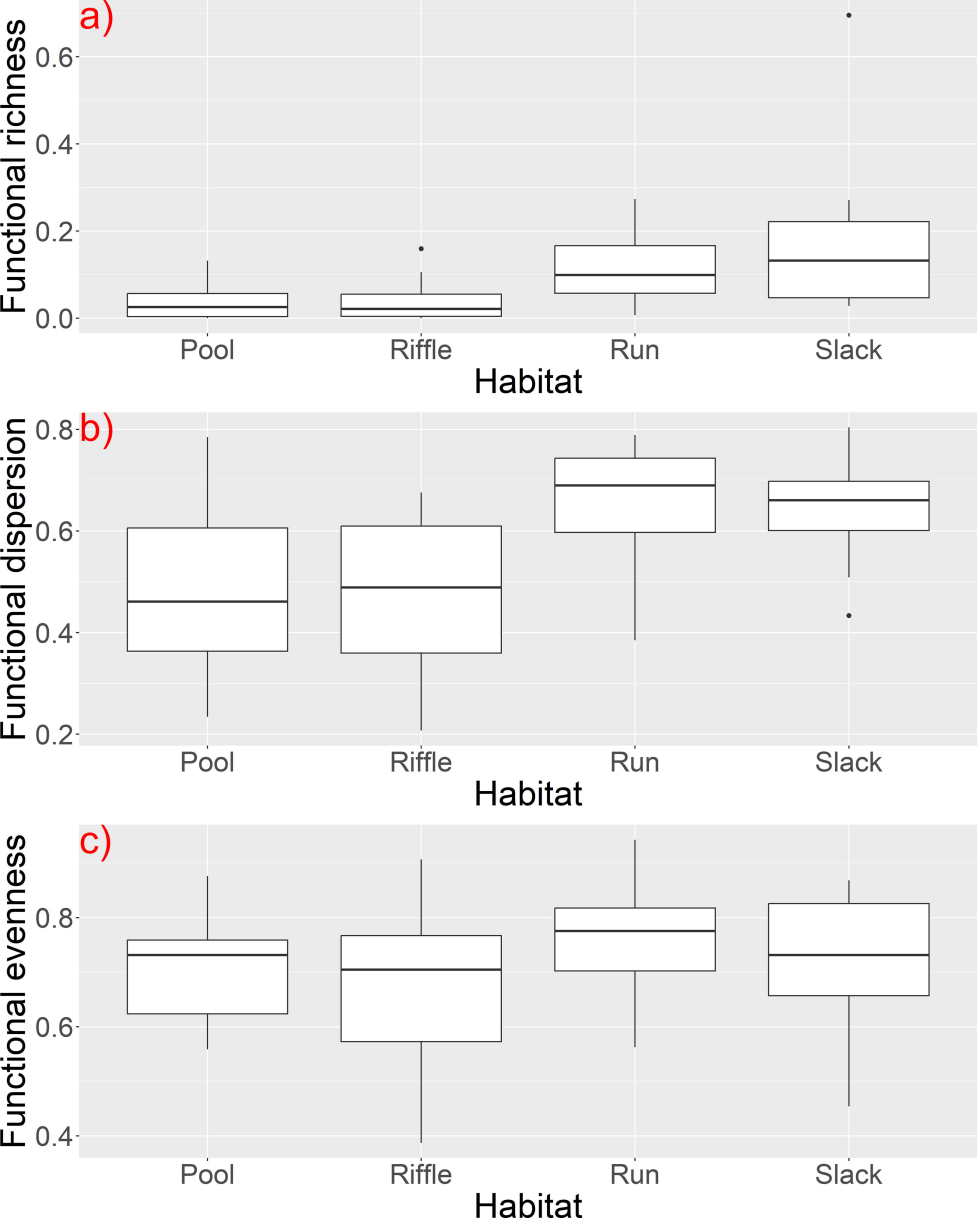
Boxplots of **(A)** functional richness, **(B)** dispersion and **(C)** evenness among river habitat types.

The results of the Kruskal-Wallis tests indicated significant variations of α functional diversity indices (**Table 5**) among the channel bed stability (stable, solid, soft, unstable) and habitat type (pool, rifle, run, slack) (**Figure 7**). In addition, functional evenness and functional dispersion showed statistically significant differences among the channel substrate and the degree of hydrological alteration respectively. Concerning the species richness, we found less significant differences (p ≤ 0.1) among the habitat type, the adjacent land uses and the effect of water abstraction (**Table 5**).

**Table 5:** Results of Kruskal-Wallis tests showing significant differences (values in bold) of the functional diversity indices among the levels of hydromorphological factors.

	Functional richness	Functional dispersion	Functional evenness	Species richness
Channel substrate	NS	NS	**P=0.002**	NS
Bed stability	**P=0.037**	**P=0.034**	**P=0.006**	NS
Shade	NS	NS	NS	NS
Habitats	**P ≤ 0.001**	**P ≤ 0.001**	**P=0.078**	**P=0.053**
Land Uses	NS	NS	NS	**P=0.067**
Channel profile alteration	NS	NS	NS	NS
Morphology alteration	NS	NS	NS	NS
Habitat alteration	NS	NS	NS	NS
Stream hydrology alteration	NS	**P=0.019**	NS	NS
Water abstraction	NS	NS	NS	**P=0.037**
Dykes (flood protection)	NS	NS	NS	NS

NS, non-significant.

The results of the generalized dissimilarity modelling showed that geographical distance was the sole significant predictor for the Sørensen functional dissimilarity, although the model explained just the 8.5% of the total deviance. The models for the components turnover and nestedness explained even lower shares of total deviance (3.9 and 2.1) with none of the environmental variables being significant. We found similar results for the taxonomic dissimilarity with geographic distance being a significant predictor for Sørensen dissimilarity and the turnover component, but both models explained 6.9 and 7.4 of the total deviance respectively. The GDM for the taxonomic nestedness explained 3.2% of the total deviance, with none of the environmental variables being significant.

PERMANOVA showed significant variations of the Sørensen functional dissimilarity and the turnover component among most hydromorphological factors (p ≤ 0.1), but with low R^2^ ranging between 0.05 and 0.15 (**Table 6**).

**Table 6:** Results of PERMANOVA showing significant variations of the functional dissimilarity matrices among the levels of hydromorphological factors.

	Total Sørensen dissimilarity	Turnover	Nestedness
Channel substrate	P=0.031, R.sq=0.04	P=0.021, R.sq=0.07	NS
Bed stability	P=0.002, R.sq=0.07	P=0.002, R.sq=0.12	NS
Shade	P=0.003, R.sq=0.07	P=0.003, R.sq=0.09	NS
Habitats	P=0.001, R.sq=0.11	P=0.001, R.sq=0.15	NS
Land Uses	NS, R.sq=0.05	P=0.099, R.sq=0.06	NS
Channel profile alteration	NS, R.sq=0.05	NS, R.sq=0.05	NS
Morphology alteration	P=0.011, R.sq=0.06	P=0.008, R.sq=0.10	NS
Habitat alteration	P=0.001, R.sq=0.08	P=0.001, R.sq=0.14	NS
Stream hydrology alteration	P=0.001, R.sq=0.08	P=0.001, R.sq=0.13	NS
Water abstraction	P=0.002, R.sq=0.08	P=0.001, R.sq=0.14	NS
Dykes (flood protection)	P=0.046, R.sq=0.05	P=0.028, R.sq=0.08	NS

R squared values are also shown. NS, non-significant.

## Discussion

The study of plant ecomorphological traits attempts to link morphological characteristics (e.g. habitus size, shape and morphology) with species functions. In this article, we assessed functional diversity patterns and changes of functional composition of aquatic macrophytic communities of riverine ecosystems in Greece. We identified notable variations of the functional composition in terms of key trait characteristics, such as life growth form and preference to light conditions. We also managed to distinguish a few significant relationships between functional diversity (alpha and functional dissimilarity) and environmental variables.

Our results indicated that growth form and light preference are key trait characteristics that grouped the aquatic macrophytes into five discrete groups based on their functional trait composition. Macrophytes were distinguished along a gradient that reveals light availability as a key driver that shapes macrophytic communities in the investigated river reaches. The PC1 has a high affinity with rooted submerged macrophytes and macrophytes with high EIV L value (which indicates preference to higher light intensity) and thus plants that were positioned at the left part of the PCA plot are those that could require waters with high clarity and undisturbed conditions (e.g. oxygenated waters and less turbidity). Previous studies have highlighted the role of light availability as an important environmental filter that restricts certain functional traits of aquatic macrophytes (Fu et al., 2014b; Su et al., 2019) and promoting others that can help plants to persist to the environmental conditions. However, when we examined the relationship between functional diversity indices and environmental variables, including water quality features that are related with light availability (e.g. turbidity and nutrient concentrations), we did not find evidence that could explain a possible effect of water clarity on functional diversity. Specifically, we did not find any significant effect of water quality parameters on functional richness, but only a significant effect of pH and oxygen saturation on both functional dispersion and evenness. Similarly, we did not find any significant effect of water quality variables on the taxonomic richness. Previous studies in Greek rivers have noted relatively moderate and high levels of nutrients in several sites, indicating signs of eutrophication (Stefanidis et al., 2021). Narrow gradients of nutrient concentrations and high occurrence of macrophytes that can be found in a wide range of trophic conditions (e.g. *Myriophyllum spicatum, Potamogeton nodosus, Stuckenia pectinata*) may explain the difficulty in finding specific patterns of responses along water quality gradients. These results are similar with those published by Zelnik et al. (2021), who also found *Myriophyllum spicatum* as the most common aquatic macrophyte species in watercourses in Slovenia, despite relatively different environmental conditions from those in Greece. Among the submerged macrophytes or macrophytes with floating leaves, the most common species were: *Myriophyllum spicatum, Potamogeton nodosus, Potamogeton perfoliatus, Elodea canadensis, Potamogeton crispus, Stuckenia pectinata*. This is another evidence for wide ecological amplitudes of these species, which make the interpretations more difficult. Furthermore, excessive nutrients are related with eutrophication processes that reduce light availability and may have substantial effects on taxonomic and functional diversity in standing waters (Stefanidis and Papastergiadou, 2019; Lindholm et al., 2020). However, water transparency in rivers can be affected by geohydromorphological factors such as erosion, sediment load, geology, land uses and rainfall intensity (Chalov and Prokopeva, 2022; Lu et al., 2023) that are not necessarily related with water chemistry (e.g. nutrients). Our findings also showed a significant differentiation of the functional diversity indices among the types of bed stability (stable, solid, soft and unstable) and the types of habitat (pool, rifle, run and slack), which suggests that hydromorphological conditions (e.g. substrate rigidness, depth and flow type) are important drivers of certain plant traits. Functional richness, dispersion and evenness were higher at slack and run habitats, which are characterized by deeper waters and slow or fast water flow respectively. All three indices were also higher at soft substrates (mostly sand, silt and mud) than more stable substrates (e.g. bedrock, gravel, cobbles and boulders). Similarly, functional evenness was higher at fine substrates (p=0.002) which confirms a possible association of increased functional diversity with soft and fine river substrates. Previous studies have shown that geomorphological features such as river bottom type, substrate structure and riverbank stability are important factors for explaining macrophyte composition in lotic ecosystems (Peternel et al., 2022). Rooted aquatic plants in particular, prefer fine sediments (Willby et al., 2000; Hrivnák et al., 2010) while bryophytes usually occur at coarser substrates, such as boulders and cobbles. Thus, life forms are differentiated among various types of substrates. Moreover, macrophytes are considered to be ecological engineers because macrophyte assemblages have a positive impact on fine sediment accumulation on the river bottom, modifying the channel bed and facilitating plant colonization (Jones et al., 2012). We did not find any other significant differentiation of α diversity indices among the remainder hydromorphological factors, except for a significant effect of hydrological alteration and water abstraction on functional dispersion and evenness respectively. Although aquatic plants are known to respond to hydromorphological changes (Szoszkiewicz et al., 2014; Baattrup-Pedersen et al., 2016; Turunen et al., 2016; Birk et al., 2020; Gyosheva et al., 2020), only a few studies have highlighted the role of hydromorphology as an important driver of aquatic macrophytic diversity, including functional diversity (Manolaki et al., 2020; Stefanidis et al., 2021; Vukov et al., 2022). Besides substrate, hydrology plays a major role in promoting species with traits that enable them to persist droughts and low flow conditions in Mediterranean rivers (Manolaki et al., 2020). In our case, we used nominal and ordinal hydromorphological variables that limited our capability to fully explore how functional diversity changes across hydromorphological gradients (e.g. hydrological alteration). Still, we were able to capture the effect of different substrates and river habitats and extract useful conclusions on the conditions that favor increased functional diversity.

Another issue that might explain why we did not find significant relationships between certain environmental parameters and diversity indices, is the intraspecific trait variability that many plants exhibit, including aquatic plants (Fu et al., 2014b). Some plants may show high phenotypic plasticity that provides them with various adaptations to large environmental changes (Fu et al., 2018; Lindholm et al., 2020). For instance, a submerged macrophyte in China, *Potamogeton maackianus*, can form large monospecific beds across wide environmental gradients (Fu et al., 2018). Similar macrophyte assemblages with a few species that occur across wide ranges of nutrients and physico-chemical gradients are common in Greece (Stefanidis et al., 2019), such as *Myriophyllum spicatum* and *Potamogeton nodosus.* Other plants can show adaptations to water level fluctuations showing various growth forms (e.g. emergent, rooted with floating-leaved plants, or rooted submerged) that can help them offset environmental limitations such as limited light availability. It is likely that the inclusion of intraspecific trait variability in trait-based studies could further elucidate the functional responses to environment gradients.

An additional finding of the current research concerns the relationship between taxonomic and functional diversity. The relationship between taxonomic and functional richness has been previously used to investigate the functional redundancy of communities (Ricotta et al., 2016; da Silva Camilo et al., 2018). In this study, we found a positive significant relationship between functional and taxonomic richness that indicates low functional redundancy. This means that the loss of a few species from the macrophyte communities is more likely to lead to loss of certain functions that are strictly related with those species. This finding is particularly important for conservation scientists and environmental managers because it emphasizes the need to include the monitoring of functional diversity (besides taxonomic diversity) in order to better assess the impact of environmental changes (Ricotta et al., 2016; Biggs et al., 2020).

Separating turnover and nestedness-resultant contributions to the overall β-diversity could provide further insights into mechanisms shaping community composition with respect to β-diversity. We found that the taxonomic β-diversity was mainly governed by turnover, i.e., replacement of disappearing species by new emerging species along the gradient, which is a typical finding in freshwater studies (Perez Rocha et al., 2019). Studies that explore the relationship between taxonomic and functional beta diversity in freshwaters are not common, and their results are rather ambiguous (Perez Rocha et al., 2019; Teittinen and Virta, 2021). In the current research, we found that functional β-diversity was higher than taxonomic β-diversity. The high functional dissimilarity may partly stem from low number of taxonomic species versus considerably higher number of functional traits or functions. A discrepancy was also revealed in the decomposition of functional β-diversity, where the higher levels of functional β-diversity were mainly due to a higher nestedness-resultant component compared to taxonomic β-diversity decomposition, where the taxonomic and functional β-diversity turnover were at similar levels. Thus, replaced species in functionally poor assemblages held traits already included in the functional space of functionally rich ones, resulting in increased functional nestedness. The results of the GDM did not show strong indications of environmental effects on the taxonomic and functional dissimilarity rate and thus our study does not provide support for the role of environmental filtering as a driver of neither functional nor community dissimilarities. The PERMANOVA results showed significant variation of the overall β-diversity and the turnover among the majority of hydromorphological factors but the R^2^ values were quite low indicating a large share of unexplained variation. It is likely that regional spatial processes (dispersal limitation with increasing geographic distance) are more important factors than local environmental descriptors for aquatic macrophyte taxonomic and functional composition changes among sites (Oikonomou and Stefanidis, 2020; Stefanidis et al., 2021).

## Conclusions

This article provides new information filling the gap of knowledge of the functional responses of aquatic macrophytic assemblages to environmental gradients in an extended network of running waters from mainland Greece. With this article we quantified the a- and β- functional diversity of aquatic macrophytic communities of river reaches and we attempted to look for significant responses to environmental parameters related with water quality gradients and hydromorphological factors. We found that the trait characteristics that contributed most to explaining the total variance of the functional space were the macrophyte growth form and the preference to light conditions which indicates that light availability plays a major role in filtering traits of aquatic plants. We did not find any clear indication of strong relationships between functional diversity and water quality gradients. We found significant variations of alpha and beta functional diversity among hydromorphological factors - mainly substrate and river habitat - which suggested that lotic systems with fine substrates and deep waters (run and slack habitats) promoted functional diversity. We consider likely that further studies to explore the effects of additional hydromorphological gradients could reveal significant responses of functional plant communities. An important finding was the positive relationship between species richness and functional richness which implies that the loss of taxonomic richness could lead to a loss of functions. Overall, our study provides useful insights and recommendations concerning the study of functional diversity of aquatic plant assemblages within the frame of freshwater monitoring and conservation.

## Data availability statement

The raw data supporting the conclusions of this article will be made available by the authors, without undue reservation.

## Author contributions

KS, AO and EP contributed to the conception and design of the study. EP, GD, MS, DT and KS contributed to data compilation. KS and AO analysed the data and wrote the manuscript. KS, AO and EP authors contributed to the final version of the manuscript.
